# Storage stability of konjac glucomannan/curdlan films at low temperature and its coating for the preservation of cucumbers

**DOI:** 10.1111/1750-3841.70094

**Published:** 2025-03-07

**Authors:** Runmiao Tian, Jun Jiang, Kao Wu, Ying Kuang, Bo Peng, Kai Chen, Fatang Jiang

**Affiliations:** ^1^ National “111" Center for Cellular Regulation and Molecular Pharmaceutics Key Laboratory of Fermentation Engineering (Ministry of Education), Hubei University of Technology Wuhan China; ^2^ Hubei Key Laboratory of Industrial Microbiology Hubei University of Technology Wuhan China; ^3^ Faculty of Engineering University of Nottingham Nottingham UK

**Keywords:** coating, cold storage stability, curdlan, konjac glucomannan, water transfer

## Abstract

Fruits and vegetables suffer severe moisture loss during cold storage. To explore the mechanism of water transfer, this study investigated the properties of konjac glucomannan (KGM)/curdlan (KC) composite films after cold storage treatment, the preservation of KC‐coated cucumbers, and the water transfer. The results showed that the weight, thickness, free water content, and enthalpy (ΔH) of endothermic peak of the film increased after cold storage, mainly because of the water adsorption and diffusion. K_6_C_4_ (the KGM/curdlan mass ratio in 6:4) maintained uniform and dense and showed the lowest dissolution loss of 21.92%. Moreover, the water content of K_6_C_4_ film changed by 1.1% on day 15, and K_6_C_4_ exhibited excellent gas barrier and mechanical properties. These were attributed to the optimal matrix formed by the assembly of KGM and curdlan in K_6_C_4_, contributing to the stability of structure and performance. K_6_C_4_ coating significantly maintained the quality of cucumbers. At the end of storage, the firmness and weight loss of the coating group were 19.3% and 24.4% higher than the control group, respectively. The color, total solid content, acid, and VC were maintained for coating group. The low‐field nuclear magnetic resonance revealed that K_6_C_4_ coating inhibited the water transfer from the center to the epidermis of cucumbers by blocking the water produced by respiration and the free water in the tissues. The storage stability and water transfer analysis will contribute to the understanding of the mechanism of coating inhibiting moisture loss of fruits and vegetables.

## 1 INTRODUCTION

Moisture loss is a common phenomenon during the storage of fruits and vegetables, resulting in water loss, wilting, shrinking, and even decay. Temperature is an important factor affecting the physiological metabolism of fruits and vegetables during storage (Ning et al., 2019). Low temperature can decrease the respiration intensity and metabolic rate. The water produced by respiration will contribute to water activity (*a*_w_) and water transfer. The water vapor pressure difference between fruits and vegetables surface and the cold storage environment contributes to the water transfer continuously. Therefore, the moisture loss of fruits and vegetables at low temperature needs to be controlled.

Edible coating is an important way for the preservation of fruits and vegetables. Coatings play as barriers to significantly inhibit the entry of surrounding oxygen and the escape of water and carbon dioxide (Maringgal et al., 2020; Zhu, 2021). Natural biopolymers like polysaccharides and proteins are the major materials for packaging (Donaldson, 2020; Yousuf et al., 2021). The chitosan/litchi peel extract/nano‐TiO_2_ coating was utilized in watercored apples, showing the lowest weight loss. Moreover, the peak respiration of the coating group was delayed until day 75. Both the low temperature (0 ± 1°C) and high relative humidity (RH) reduced moisture loss (Liu et al., 2021). The storage period of cucumbers covered by starch glucose coating was extended up to 30 days under refrigerated temperature (Patel & Panigrahi, 2019). During cold storage, the physicochemical properties of the films/coatings will be affected by temperature and change with time. The carboxymethylcellulose/gelatin films were investigated by atomic force microscopy. The results showed that the roughness increased after 12 days at 2–8°C and −20°C and the height difference was greater at frozen conditions for adsorbing more moisture (He et al., 2022). During the storage of soy protein isolate films at 25°C, 4°C, and −18°C, a large number of voids and cracks were found in their cross‐section after 6 weeks (Zhang et al., 2021). The microstructure and properties of some coatings were not damaged by low temperature (He et al., 2022; Shahrampour et al., 2020). However, the moisture content of the coatings might change and affect the intermolecular channels as time goes by.

Based on our previous research, the konjac glucomannan (KGM)/curdlan (CUD) (KC) coatings significantly maintained the quality of cherry tomatoes at room temperature (Chen et al., 2023a). During film formation, hydrogen bond interactions and weak electrostatic interactions existed between the KGM and curdlan molecules. And hydrophobic interactions were also formed within the methylene groups of curdlan (Ye et al., 2019). Under the effects of these interactions, uniform and dense films were formed. KGM is extracted from konjac tubers and possesses the properties of edibility, biocompatibility, degradability, and film formation. It has been widely applied in the food industry, such as food packaging (Chen et al., 2023b; Wu et al., 2023). The KGM‐based composite coating with gallic acid extended banana shelf‐life to 7 days (Deng et al., 2024). The polysaccharide CUD, from non‐pathogenic bacteria, has a special three‐helix molecular structure. It has been widely used as an edible packaging material due to its unique gel properties, non‐toxicity, biodegradability, and film formation (Yuan et al., 2021). The addition of CUD significantly improved the elongation at break (EAB) and water barrier of chitosan coating. When the mass ratio of chitosan and CUD was 1:1, the composite coating was significantly better than single‐component coating in preserving cherry tomatoes (Yu et al., 2024). This study aims to clarify the storage stability of KC films and the preservation effect of its coating on cucumbers, as well as the water transfer in the cold storage environment/KC coating/cucumbers (ECC) system. This work will benefit the understanding of the mechanism of edible coatings inhibiting the moisture loss of fruits and vegetables at low temperature.

## 2 MATERIALS AND METHODS

### 2.1 Materials

KGM was obtained from Wuhan Licheng Biotechnology Co., Ltd. and purified by ethanol solution method (Niu et al., 2023). Curdlan (purity ≥ 95%) was purchased from Shandong Zhongke Biotechnology Co., Ltd. Cucumbers (*Cucumis sativus* L.) of uniform size and maturity were purchased from the local market (Wuhan, China; 30°28′ N, 114°18′ E), without mechanical damage. Cucumbers were harvested and bought for immediate experimental use.

### 2.2 Preparation of KGM/CUD composite films

The films were prepared by using a casting method. The KGM solution was made by dissolving KGM powder (the amount varying with the ratio of the following composite solutions) evenly into 75 mL water and continuously stirred at 600 rpm and 60°C for 1 h. Curdlan powder was dispersed in water (25 mL) at 25°C for 30 min and continuously stirred for 30 min at 60°C to obtain curdlan dispersion. Next, the curdlan dispersion was mixed with KGM solution and stirred for 1 h at 900 rpm and 90°C. The resulting composite solution was used to prepare subsequent films and coatings. To mimic the thickness and formation of the coating, 30 ± 0.05 g of film‐forming solution was cast on the plastic plate (15‐cm diameter) and dried at 25°C in the oven. The total solid content of the film‐forming solution was kept at 1% (w/v), and the ratios of KGM/curdlan were as follows: 10:0, 8:2, 6:4, 4:6, 2:8, 0:10 (w/w). The films were coded as KGM, K_8_C_2_, K_6_C_4_, K_4_C_6_, K_2_C_8_, and Curdlan.

### 2.3 Cold storage treatment of KC films

The KC films were put in commercial fruit and vegetable crispers (250 mm × 167 mm × 91 mm) and stored in a domestic refrigerator for 15 days. In particular, the refrigerator was set at 4°C and 60% RH (simulating the conditions of a home refrigerator). The temperature and RH of the storage environment were monitored by using a recorder (COS‐03, Jianda Renke Electronic Technology Co., Ltd). The characterizations of the films were examined by sampling on day 0 and 15.

### 2.4 Characterizations of the KC films at low temperature

#### 2.4.1 Weight and thickness changes

The weight of the films (5 × 10 cm) was obtained by an electronic balance (ME204/02, Mettler Toledo Co., Ltd.) at the beginning and end of the treatment. The weight change ratio was calculated using Equation (1).

The thickness was determined with a digital micrometer (Dongguan Sanliang Measuring Tools Co., Ltd.) at the beginning and end of the treatment. Ten points were randomly determined for each film. The thickness change ratio was calculated using the following Equation (2).

(1) $$\mathrm{Weight}\;\mathrm{change}\;\mathrm{ratio}\;(\%)=\frac{w_{t}\;-\;w_{1}}{w_{1}}\;\times \;100\%$$

where *w*_1_ (g) is the initial weight of the films and *w*_t_ (g) is the weight on day 15.

(2) $$\mathrm{Thickness}\;\mathrm{change}\;\mathrm{ratio}\;(\%)=\frac{T_{t}\;-\;T_{1}}{T_{1}}\;\times \;100\%$$

where *T*_1_ (µm) is the initial thickness of the films and *T*_t_ (µm) is the thickness on day 15.

#### 2.4.2 Moisture content

The films were sampled (3 cm × 4 cm) after the treatment of cold storage and dried at 105°C for 1 h in an oven (Jinghong Laboratory Instrument Co., Ltd.), and then weighed. The moisture content was calculated using the following Equation (3).

(3) $$M\;(\%)=\frac{m_{0}\;-\;m_{t}}{m_{0}}\;\times \;100\%$$

where *M* is the moisture content, *m*_0_ (g) is the weight of the sample on day 15 before drying, and *m*_t_ (g) is the weight of the sample on day 15 after drying.

#### 2.4.3 Attenuated total reflectance‐Fourier transform infrared spectroscopy (ATR‐FTIR)

According to the previous method (Chen et al., 2023a), the spectrograms of the films stored for 15 days were obtained using an ATR‐FTIR spectrometer (Nicolet iS10, Nicolet Co. Ltd.) in the wavelength range of 500–4000 cm^−1^.

#### 2.4.4 Differential scanning calorimetry (DSC)

The thermal property of the films was tested using a differential scanning calorimeter (Mettler Toledo Co. Ltd.). The samples were sealed in a Mettler dish and heated from 25°C to 200°C at a rate of 10°C/min under high‐purity N_2_ environment.

#### 2.4.5 Soluble solid loss ratio

The soluble solid loss ratio was determined according to a previous method (Bu et al., 2023), which was measured after the treatment of cold storage. The films with the size of 3 × 4 cm were immersed in 100 mL water at 25°C for 0.5 h. Then the wet samples were taken out and dried at 105°C for 1 h. The soluble solid loss ratio was calculated by the following Equation (4).

(4) $$S\;(\%)=\frac{s_{1}\;-\;s_{2}}{s_{1}}\;\times \;100\%$$

where *S* represents the soluble solid loss ratio, *s*_1_ (mg) represents the dry weight of the samples on day 15 before immersion, and *s*_2_ (mg) represents the dry weight of the samples on day 15 after immersion.

#### 2.4.6 Water contact angle (WCA)

The value of the WCA on the surface of the films was determined according to the previous method (Tian et al., 2024), which was tested by a contact angle meter (OCA20, DATA PHYSICS Co., Ltd.).

#### 2.4.7 Mechanical properties

The mechanical properties of the films were evaluated after cold storage (Tian et al., 2024). Referring to the previous method, the tensile strength (TS) and EAB of the films were determined by using a Texture Analyzer (TA. XT Plus).

#### 2.4.8 Water vapor permeability (WVP)

The water vapor permeability of the films after the treatment of cold storage was tested in triplicate by a WVP tester (Stable Micro System, Co. Ltd.). The test cups filled with deionized water were sealed with the films and then put into a chamber (25°C and 90% RH).

#### 2.4.9 Oxygen permeability (O_2_P)

According to the previous method, the oxygen permeability was determined with a simple modification (Xiao et al., 2022). The specific method is as follows. First, a plastic cup of 60 mL was filled with 30 mL of soybean oil. Second, the films after the treatment of cold storage were used to seal the cup and placed at 25°C and 60% RH for 1 week. The cups without sealing served as control. Finally, the peroxide value (PV) of the oils was detected by sodium thiosulfate titration. The oxidation degree of the grease reflects the oxygen permeability of the films.

#### 2.4.10 Carbon dioxide permeability (CO_2_P)

The carbon dioxide permeability of the films was performed by the strong alkali absorption method with some modifications (Zhou et al., 2023). A plastic cup of 60 mL was filled with 30 mL of saturated potassium hydroxide (KOH) solution and was sealed with the films that had been stored at low temperature for 15 days. Each test system above was weighed and placed in a vacuum desiccator, then carbon dioxide was injected into the desiccator for 30 min. The weight of each test system was measured after 3 days. The carbon dioxide permeability was calculated by the following Equation (5).

(5) $$CO_{2}P\;(g/(m^{2}\cdot h))=\frac{\Delta m}{A\;\times \;t}$$

where Δm (g) represents the increase in weight, A (m^2^) represents the area of the mouth of the cup, which is 0.005539 m^2^, and t (h) represents the testing time.

#### 2.4.11 Scanning electron microscopy (SEM)

To observe the cold storage stability of the microstructure of the KC films, the cross‐section of the film samples on days 0 and 15 was observed by SEM (JSM‐6390LV, JEOL Ltd.). The films were broken after freezing in liquid nitrogen, and the samples obtained were sputtered with gold under vacuum for 90 s and observed at 15 kV with a magnification of 2000×.

#### 2.4.12 Low‐field nuclear magnetic resonance (LF‐NMR)

To analyze the water distribution and binding in the film, the K_6_C_4_ film with the best storage stability was selected for the measurement of LF‐NMR. The relaxation of the film on days 0 and 15 was performed by LF‐NMR spectrometer (NMI20‐040V‐I, Suzhou Niumag Analytical Instrument Co., Ltd.). The K_6_C_4_ film of uniform quality was placed in the NMR glass tube and put into the magnetic chamber at 30°C. The Carr‐Purcell‐Meiboom‐Gill (CPMG) pulse sequences were used to detect the transverse relaxation time *T*_2_. The parameters were set as follows: spectral width (SW) = 200 kHz, spectrometer frequency (SF) = 20 MHz, time waiting (TW) = 2000 ms, time echo (TE) = 0.15 ms, and number of scans (NS) = 6.

### 2.5 Preservation experiment of cucumbers

#### 2.5.1 Coating treatment of cucumbers

Cucumbers were dipped in the K_6_C_4_ solution for 1 min and then dried naturally to form a coating on the surface. The control group was dipped in water and similarly air‐dried. The number of cucumbers in each treatment was 40. All treated fruits were put into commercial fruit and vegetable crispers (250 mm × 167 mm × 91 mm) and stored at 4°C and RH 60% for 15 days. The preservation effect of the KC coating was investigated on days 0, 3, 6, 9, 12, and 15.

#### 2.5.2 Weight loss

The treated cucumbers on days 0, 3, 6, 9, 12, and 15 were weighed, and the weight loss ratio was calculated the following Equation (6).

(6) $$\mathrm{Weight}\;\mathrm{loss}\;\;\left(\%\right)=\frac{W_{0}\;-\;W_{t}}{W_{0}}\;\times \;100\%$$

where *W*_0_ is the initial weight of cucumbers (g) and *W*_t_ is the weight at different storage times.

#### 2.5.3 Firmness

The firmness of the cucumbers every 3 days was determined using a texture analyzer (TA. XT. plus, Stable Micro System Co., Ltd.) with a p/2 probe of 2‐mm diameter. The pre‐test speed, test speed, and post‐test speed were set as 10, 0.5, and 10 mm/s, respectively. In addition, the depth of the probe entering the fruits was 6 mm. Each sample was measured five times.

#### 2.5.4 Skin color

The skin color of the cucumbers was determined by a precise color reader (WF28, Shenzhen Wave Optoelectronics Technology Co., Ltd.). The L* (brightness), a* (red or green), and b* (yellow or blue) were obtained by the color reader. Skin color differences (ΔE) were calculated using the following Equation (7).

(7) $$\Delta E=\sqrt{{\left(L^{*}-L_{0}\right)}^{2}+{\left(a^{*}-a_{0}\right)}^{2\;}+{\left(b^{*}-b_{0}\right)}^{2}}$$

where L*, a*, and b* represent the measured values of cucumbers on day t (*t* = 0, 3, 6, 9, 12, and 15), and *L*_0_, *a*_0_, and *b*_0_ represent the initial values on day 0.

#### 2.5.5 Total solid content and total acid

The total solid content and total acid were measured using a sugar acid machine (PLA‐BXIACID1, ATAGO Ltd.). Cucumber juice was dropped on the test platform to obtain the total solid content value. To test the total acid, pristine cucumber juice was diluted 50 times to be detected. The machine should be zero‐set with pure water before use.

#### 2.5.6 Ascorbic acid

According to the previous method with modifications (Chen et al., 2023a), the spectrophotometric (TU‐1900, Purkinje General Instrument Co., Ltd.) was used to analyze the ascorbic acid concentrations of the cucumbers every 3 days.

#### 2.5.7 LF‐NMR of cucumbers

The LF‐NMR detection is the same as that of the film. The treated cucumbers on days 0, 5, 10, and 15 were cut into slices along the cross‐section with a thickness of 1 cm. The parameters set for the CPMG experiment were as follows: SW = 100 kHz, SF = 20 MHz, TW = 5000 ms, TE = 1 ms, and NS = 8. Both the coated and control groups were measured in triplicate. The water distribution of cucumbers was also characterized by magnetic resonance imaging (MRI). The pulse parameters were as follows: TE = 20 ms, successive scans = 500 ms, scan repetitions = 256, and averages = 16.

### 2.6 Statistics

Every measurement was conducted in triplicate or more. All data were subject to the SPSS statistics software and compared among groups by one‐way analysis of variance with the Duncan multiple range test (*p *< 0.05). Moreover, graph drawing was carried out by Origin 2018.

## 3 RESULTS AND DISCUSSION

### 3.1 Changes in the weight and thickness of KC films

**FIGURE 1:**
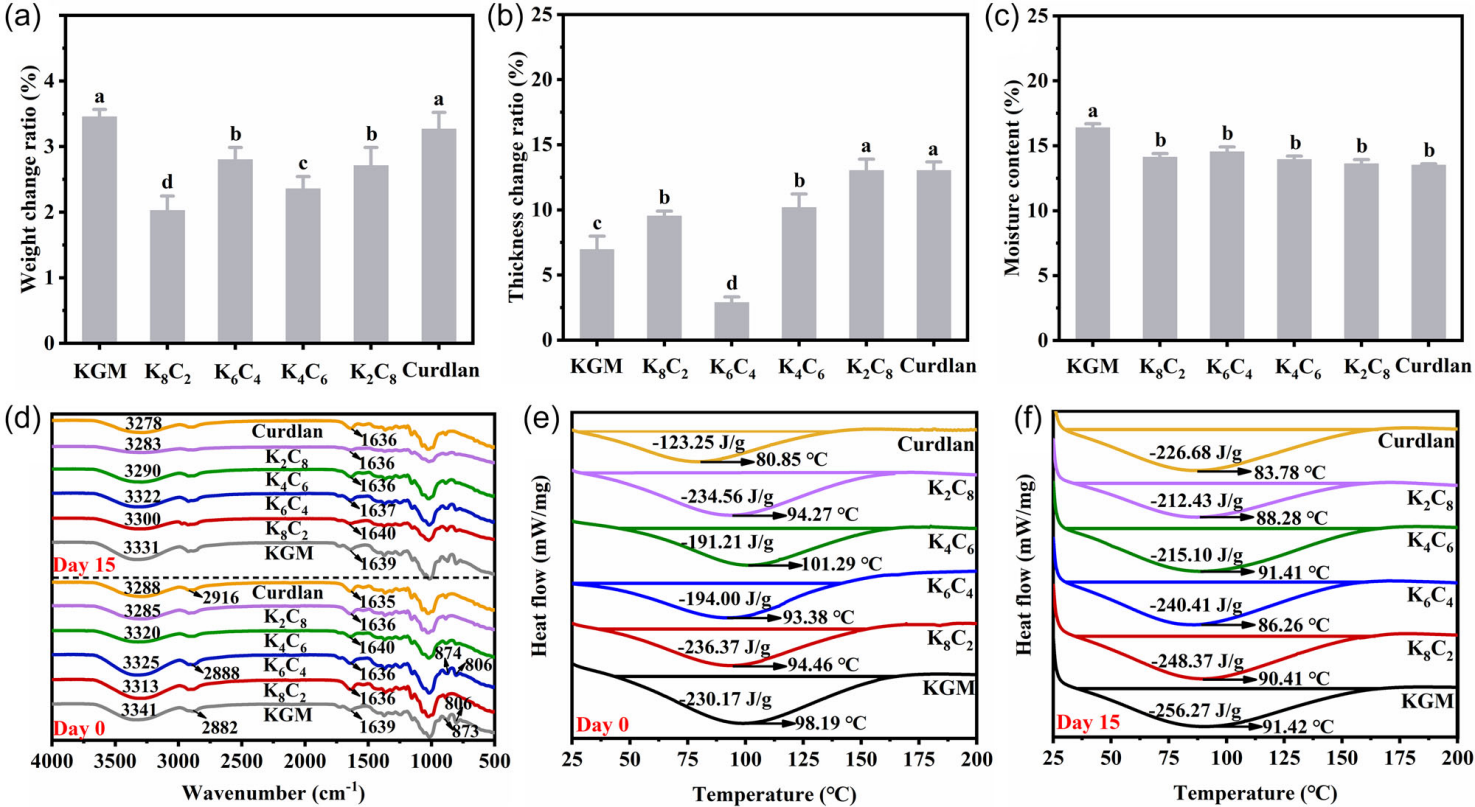
The weight change ratio (a), thickness change ratio (b), moisture content (c), Fourier transform infrared spectroscopy (FTIR) spectra (d), and differential scanning calorimetry (DSC) curves (e and f) of konjac glucomannan (KGM)/curdlan (CUD) (KC) films after cold storage treatment. KGM, konjac glucomannan.

As a barrier layer between fruits and vegetables and the storage environment, the transfer of internal and external water takes place in the films, including adsorption, diffusion, combination, evaporation, penetrating, etc. (Danov et al., 2021), so it is necessary to detect the weight change of the films during cold storage. The detection of weight change facilitates the analysis of the water transfer in the films. The weight change ratio of the KC films after the treatment of cold storage is shown in Figure 1a. Overall, the weight of each film was increased after the treatment, which would provide a moisturizing surface for the fruits and vegetables. At the end of the storage, the weight of each film increased to >2%. This indicated that some water molecules in the cold storage environment were adsorbed by the films, which should be attributed to the hydrophilicity of the KC films and the high RH of the storage environment (Wu et al., 2022). The adsorption of water by the films was related to water activity. Kruk et al. (2023) revealed that water in furcellaran/chitosan/carp skin gelatin hydrolysates composite films presented a non‐bound or free state when *a*_w_ was greater than 0.53, and the films would absorb less water at lower *a*_w_. In addition, as shown in Figure 1a, the addition of CUD led to a decrease in the weight change of the films, which was attributed to an increase in hydrophobicity. Among the KC composite films, the weight of K_6_C_4_ changed the most, which might be because the molecular chains of KGM and CUD form an optimal matrix, providing a more favorable diffusion for adsorbed water.

The film thickness can reflect the molecular interaction and the density of the matrix in the film‐forming process, as well as being related to mechanical properties, water adsorption capacity, and WVP (Brodnjak, 2017). In this study, the changes in film thickness during cold storage reflected the interaction between the KC films and the water from the environment and the transfer of water. As can be seen in the thickness change ratio in Figure 1b, the thickness of all films was increased compared to the initial value. This could be explained by the fact that the films absorbed water from the environment with the subsequent water diffusion, and part of the water tended to fill in the polysaccharide matrix or gather around the KGM and curdlan molecules, which caused the film matrix to stretch, increasing thickness (Zhu, 2014). This result was in accordance with the weight change of the films. It was worth noting that the thickness of the K_6_C_4_ film increased the least, approximately 2.5%, while that of K_2_C_8_ reached the highest. These indicated that KGM and curdlan in K_6_C_4_ film had the best miscibility and structural stability to form a composite film that was not easily infiltrated by water, which was consistent with our previous study that K_6_C_4_ film possessed the lowest WVP (Chen et al., 2023a). Moreover, high curdlan content, such as K_2_C_8_ and pure curdlan films, showed a high thickness change ratio, probably due to their looser molecular assembly being easily invaded by water.

### 3.2 Moisture content of KC films

The investigation of moisture content can indicate the moisture adsorption capability of the KC films during cold storage (Wu et al., 2021). As shown in Figure 1c, pure KGM film showed the highest moisture content, and pure curdlan film had the lowest one, representing the highest and lowest moisture adsorption capacity, respectively, which depended on their hydrophilicity, supporting our previous study (Wu et al., 2022). KC composite films exhibited the middle value of moisture content. The addition of CUD led to a decrease in moisture adsorption capability, and this probably was caused by the reduced solid percentage of hydrophilic KGM components in the films, which allowed easier moisture adsorption. Additionally, the K_6_C_4_ film, with a slightly higher moisture content than the other KC films, indicated the formation of an optimal matrix suitable for the diffusion of water, in agreement with the results of weight change. We could assume the water transfer during storage. A high concentration of hydrophilic KGM would facilitate the water adsorption on the surface of the films and then induce a slow diffusion in the interior. The strong hydrophilicity of KGM pushed the transfer of water molecules to the starch matrix at high RH (Chen et al., 2008).

### 3.3 FTIR spectrum of KC films

According to the FTIR spectrum, the characteristic peaks of the films before and after storage were similar, indicating that the low‐temperature treatment did not change the molecular structure of the film matrix (Figure 1d). The characteristic peaks about KGM and CUD have been reported in previous study (Chen et al., 2023a). The broad peak around 3300 cm^−1^ was attributed to the stretching vibration of −OH and intermolecular hydrogen bonding. The absorption peak around 1635–1640 cm^−1^ was the bending vibration of water molecules. For the absorption peak around 3300 cm^−1^, there was a shift toward lower wave numbers after storage compared to day 0. This showed an increase in −OH group stretching vibration and an increase in intermolecular hydrogen bonding in the film. Due to the hydrophilicity of the film matrix and the high humidity of the storage environment, water molecules were bound to the film. This supported the phenomenon of increasing film weight and moisture content after storage. For the absorption peak around 1636 cm^−1^, the peak position did not change significantly before and after storage. Xiao et al. (2012) investigated the interaction of polysaccharide molecules with water molecules in the O–H stretching region (3700–3000 cm^−1^) by using FTIR. The results showed that the absorption peak at 3420 cm^−1^ was attributed to water molecules hydrogen bonds between water and polar groups of polysaccharides or other water molecules and the intensity of the absorption peak at 3420 cm^−1^ increased with increasing alginate content.

### 3.4 DSC of KC films

The changes in thermal stability of the films before and after storage were analyzed by DSC (Figure 1e,f). In the range 25°C–200°C, all film samples had a single endothermic peak (80°C–102°C), which was produced by the evaporation of a little water from the film with the hot air flow. The peak temperature is the glass transition temperature (*T*g,°C) of the sample. The size of the peak depends on the moisture content of the sample and the interaction degree between the film components (Mouzakitis et al., 2022). The enthalpy (ΔH, J/g) of the samples increased and *T*g decreased after storage for 15 days as compared to the un‐stored samples, indicating that water molecules in the environment were adsorbed onto the film. Due to the water plasticizing effect (Kibar & Us, 2013), the presence of water molecules weakened the force between KGM and CUD and increased the mobility of polysaccharide chains. Similarly, Zein films with high moisture content had lower *T*g and larger heat absorption peak. And the *T*g of zein‐based films gradually decreased with the increase of moisture content and sunflower oil content (Mouzakitis et al., 2022).

### 3.5 Soluble solid loss ratio and WCA of KC films

**FIGURE 2:**
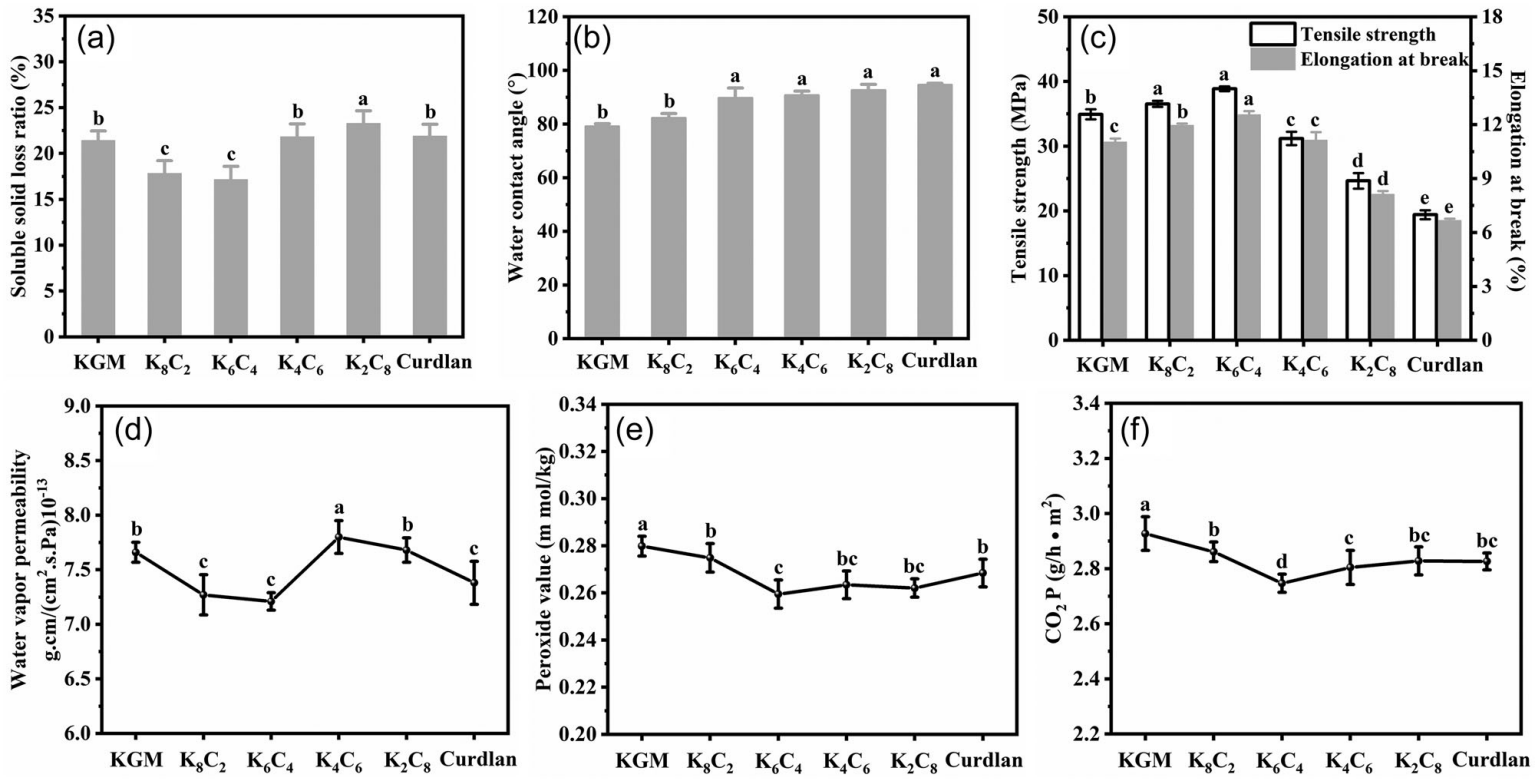
The soluble solid loss ratio (a), water contact angle (b), mechanical properties (c), water vapor permeability (WVP) (d), O_2_P (e), and CO_2_P (f) of the konjac glucomannan (KGM)/curdlan (CUD) (KC) films after cold storage treatment. KGM, konjac glucomannan.

The determination of the soluble solid loss ratio of KC films can contribute to the investigations of the stability of KC films during cold storage, the interaction between polysaccharides and water, and the degree of binding between polysaccharide molecules. This can benefit the subsequent application of KC coating in fruit and vegetable preservation. The entry of water molecules into the interior of the films enhanced the hydrogen bonding between the components, which was responsible for the reduction of free molecules and thus decreased the soluble solid loss ratio (Cui et al., 2023). The soluble solid loss ratio of the films after cold storage treatment can be seen in Figure 2a. The K_6_C_4_ film exhibited the lowest soluble solid loss ratio, which might be attributed to the most stable matrix formed by the assembly of KGM and CUD and indicated its excellent structural stability. This was consistent with the above results. In addition, the infiltration and diffusion of the adsorbed water caused the stretching and interaction of polysaccharides, resulting in a stronger bond between polysaccharides. The pure curdlan, K_2_C_8_, and K_4_C_6_ films showed significantly high soluble solid loss ratios due to the increase in curdlan (less hydrophilic than KGM) content, meaning an increase in free molecules.

The surface wettability of the films stored for 15 days was assessed by determining the WCA. The WCA values of KC films ranged from 79 to 94° (Figure 2b) and increased with CUD content increasing. Similarly, the WCA of methyl cellulose/curdlan composite films gradually increased with the increase in curdlan addition. This was caused by the tight triple helix structure and more hydrophobic cavities of CUD (Zhang et al., 2023). The film surface was relatively hydrophobic for WCA values greater than 90°. If the film was too hydrophilic, the applications in food packaging may be limited (Wu et al., 2022). The WCA value of K_6_C_4_ film was 89.73°, which indicated that the film was still viable for application after cold storage for 15 days.

### 3.6 Mechanical properties of KC films

The mechanical properties of the films are typically characterized by TS and EAB, where a larger TS indicated stronger rigidity and a larger EAB indicated stronger toughness of the films. Figure 2c shows that with the addition of CUD, both TS and EAB showed the trend of increasing and then decreasing. The addition of CUD enhanced the interaction between KGM and CUD, and the polysaccharide molecules interacted more closely with each other, resulting in an increase in TS. The film had a larger EAB when the KGM content was higher than the CUD content. Based on the results of moisture content, weight, and DSC, it was known that the films with high KGM content adsorbed more water molecules. Due to the plasticization effect, the intermolecular interactions between the film components were weakened, and the free volume increased, leading to an increase of film flexibility (Kibar & Us, 2013). It was shown that the mechanical properties of the films were strongly affected by the moisture content or the RH of the surrounding environment. The TS of alginate film decreased and EAB increased with increasing RH (Olivas & Barbosa‐Cánovas, 2007). The same phenomenon was observed for the regenerated cellulose‐based film. As the *a*_w_ increase, both TS and Young's modulus were decreased, and EAB was increased from 5.88% to 19.85% (Cazón et al., 2020). Water molecules provided the lubrication effect and relieved the rearrangement of the polymer chains and enhanced the resistance to rupture.

### 3.7 Gas permeability of KC films

#### 3.7.1 WVP analysis

Exploring the gas permeability of the films at the end of cold storage treatment can characterize the stability of the films in fresh‐keeping applications, including WVP, O_2_P, CO_2_P, etc. WVP is one of the most important indexes to measure the water vapor barrier property of biopolymer films applied for fruit and vegetable preservation. The WVP of KC films obtained is shown in Figure 2d. It indicated that the addition of curdlan could improve the WVP, and K_6_C_4_ possessed the lowest WVP. This indicated that K_6_C_4_ retained water barrier stability after cold storage, which was attributed to the tight binding of KGM and CUD molecular chains and the increase of hydrophobicity caused by the addition of CUD. However, the continuous addition of CUD resulted in a looser matrix of K_4_C_6_ and a significant increase in WVP. Regarding K_2_C_8_ and CUD films, the high content of CUD enhanced the hydrophobicity of the films and caused the decrease of WVP. Generally, the WVP depends on the diffusivity and solubility of water molecules in the film matrix (Souza et al., 2015). Hydrogen bonding between water and the hydrophilic groups of biopolymers weakened the interactions between KGM and curdlan, which led to the water barrier decreasing. As a result, the microstructure of the films became loose, which made it easier for water molecules to transfer through the films. Artharn et al. (2009) reported that the hydrophilicity of the film‐forming matrix significantly affected the water vapor permeability of the films, and the WVP of round scad protein‐based films with 40% chitosan addition was significantly increased.

#### 3.7.2 O_2_P and CO_2_P analysis

The respiration and physiological metabolism based on the oxygen of fruits and vegetables continue after harvesting. Carbon dioxide is one of the products of respiration, and the accumulation of carbon dioxide between the films and the fruits and vegetables will gradually inhibit respiration. Thus, the determination of oxygen and carbon dioxide barrier in the films was essential during cold storage (Wang & Long, 2014). The PV has been used as an effective mean to detect the oxygen permeability of the films. As shown in Figure 2e,f, with the addition of CUD, the barrier properties of KC films to O_2_ and CO_2_ were significantly improved. K_6_C_4_ exhibited a very low PV and the lowest CO_2_P, which was related to its optimal structure. However, the high content of CUD resulted in a looser matrix that was not conducive to the O_2_ and CO_2_ barrier.

### 3.8 Microstructure stability

**FIGURE 3:**
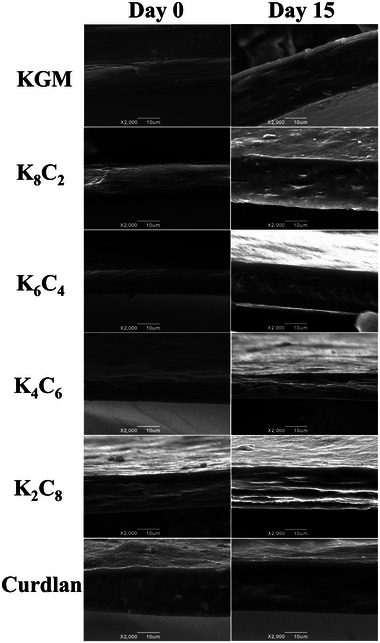
The scanning electron microscopy (SEM) photographs of the cross‐section of konjac glucomannan (KGM)/curdlan (CUD) (KC) films before and after cold storage treatment. KGM, konjac glucomannan.

The SEM photographs of the cross‐section of the KC films at the beginning and end of cold storage are illustrated in Figure 3. As a whole, no significant damage was observed in all films, indicating that cold storage would not produce obvious damage to the microstructure of the KC films. That means that edible films or coatings displayed good feasibility in the cold storage of fruits and vegetables. However, the cross‐section of the films changed by varying degrees after 15 days. Compared to day 0, the cross‐section of pure KGM, K_8_C_2_, and K_4_C_6_ films on day 15 was no longer smooth and even, with K_2_C_8_ even delaminated. Pure KGM and curdlan films exhibited a bloated texture after absorbing water. In contrast, the K_6_C_4_ film appeared to be homogeneous and dense. These results suggested that the molecular assembly of KGM and curdlan could resist the erosion of surrounding water at low temperature. Water adsorption and diffusion might be one of the reasons why some films were no longer smooth. Due to the presence of a large number of hydrophilic groups, excess water molecules would destroy the dense structure of the films, resulting in more water molecules passing through the film matrix (Song, 2022). The study revealed that water in the environment moved to the furcellaran/chitosan/carp skin gelatin films and might bind to the molecular chains and fill the pores between the molecular chains at high water activity, thus influencing the microstructure of the films (Kruk et al., 2023). Analogously, due to the occurrence of water transfer, the cavities on the carboxymethyl cellulose/gelatin blend film deepened significantly, and the roughness increased with the longer storage time. However, the change in the roughness of the films was not obvious at the later stage (He et al., 2022).

### 3.9 LF‐NMR analysis

**FIGURE 4:**
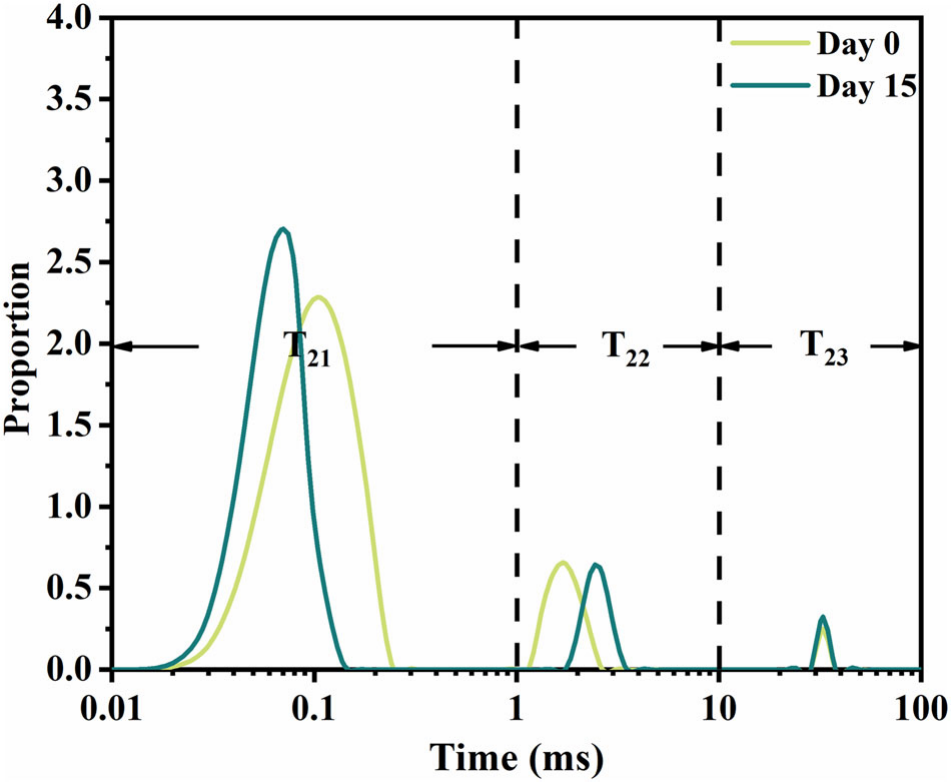
The transverse relaxation time (*T*_21_, *T*_22_, and *T*_23_) of water in K_6_C_4_ film at the beginning and end of cold storage treatment.

Water distribution and binding behavior of the K_6_C_4_ film during cold storage were determined by LF‐NMR spectroscopy and described in Figure 4 and Table 1. Three peaks appeared in the lateral relaxation time profile of the film. Transverse relaxation time (*T*_2_) was the binding degree between water, and it could be divided into three parts: *T*_21_ (0.01–1 ms), *T*_22_ (1–10 ms), and *T*_23_ (10–100 ms). *T*_21_ and *T*_22_ represented bound water and loosely bound water, respectively, which was primarily caused by the hydrogen bonds formed between groups in the molecular chain of the film matrix and water molecules (Zhang et al., 2021). *T*_23_ represented free water, and it was the water that remained after the film was dried, which was bound to the surface and inside of the film by extremely weak bonds (Zeng et al., 2022). Table 1 shows the percentage of each water type. It could be seen that the K_6_C_4_ film had the highest content of bound water throughout the cold storage and contained a small portion of loosely bound water and free water. After 15 days, the proportion of bound water and loosely bound water all showed an overall decreasing trend, while free water increased. This was mainly caused by the adsorption of water molecules to the film, resulting in an increase in the proportion of free water and a subsequent decrease in the proportion of bound water. Combined with the moisture content and weight change analysis, it was indicated that the increase in free water was mainly due to the adsorption of moisture from the external environment. The enhancement of the stretching vibration of −OH and the increase of the enthalpy of endothermic peak in DSC proved that more water was adsorbed into the film.

**TABLE 1:** The water proportions (*A*_21_, *A*_22_, and *A*_23_) of K_6_C_4_ film during cold storage.

Storage time (days)	*A*_21_ (%)	*A*_22_ (%)	*A*_23_ (%)
0	93.9700 ± 5.1374a	4.8310 ± 0.1004a	1.1990 ± 0.2451b
15	93.7700 ± 6.9422a	4.2530 ± 0.5019ab	1.977 ± 0.2848a

*Note*: Values are means ± standard deviation of three replicates. Different lowercase letters represent the significant differences within groups (*p* < 0.05). *A*_21_, *A*_22_, and *A*_23_ represent the water proportions of bound water, loosely bound water, and free water, respectively.

### 3.10 Coating for the preservation of cucumbers

#### 3.10.1 Weight loss and firmness

**FIGURE 5:**
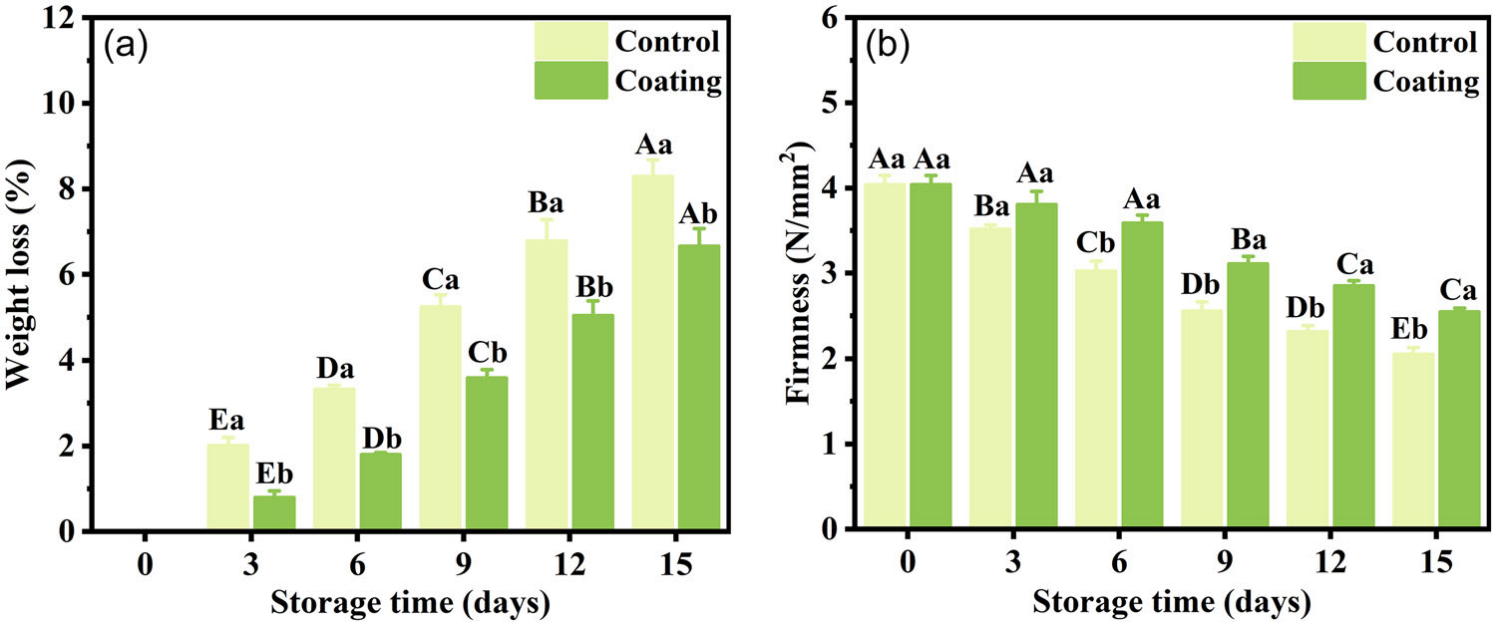
The weight loss (a) and firmness (b) of cucumbers during cold storage. Different uppercase letters (A–E) represent the significant differences within groups (*p* < 0.05), and different lowercase letters (a–b) represent the significant differences between groups at the same time (*p* < 0.05).

The transpiration and respiration of the fruits during storage lead to a wilting phenomenon and a decrease in the postharvest quality of the fruits (Patel & Panigrahi, 2019). The consumption of nutrients and evaporation of water cause the fruits to suffer weight loss. As shown in Figure 5a, the weight loss of cucumbers increased in both groups during cold storage. However, the weight loss in the coated group was significantly less than that in the control group. At the end of storage, K_6_C_4_ coating reduced the weight loss of cucumbers by 24.4%, compared to the control group. The weight loss of the coating group on day 15 was slightly lower than that of the control group on day 12, suggesting that K_6_C_4_ coating delayed the weight loss of cucumbers by >3 days. The apparent increase in weight loss in the uncoated group was attributed to the direct exposure of the fruits to the external environment thus accelerating water loss. This indicated that the K_6_C_4_ coating played a key role in preventing water from escaping in the cucumbers. Meanwhile, the coating blocked the entry of oxygen, thereby inhibiting respiration metabolism (Jiang et al., 2021).

Fruit softening was associated with pectin depolymerization and cellulase activity (Zhang et al., 2023). The degradation of cell walls and polysaccharides was directly responsible for the loss of cucumber texture (Guo et al., 2022). During the storage, the firmness of both the coated and control groups showed a decreasing trend (Figure 5b). However, the firmness of the treated cucumber was significantly greater than that of the control group. The coating is a barrier to block the permeation of oxygen and carbon dioxide. Freshly harvested cucumbers had a firmness of 4.04 N/mm^2^. After 15 days of storage, the loss of firmness in the control group was around 49%, while the coated group lost 38%. As a result, the respiration rate and the cell wall disintegration were reduced, thus delaying the fruit's firmness loss. In addition, excessive water loss from the fruits can lead to a loss of cell turgor pressure, resulting in a rapid decrease in fruit firmness (Sarker et al., 2021).

#### 3.10.2 Skin color

Color is an important indicator of the maturity and shelf‐life of cucumbers. Changes in the skin color of cucumbers during storage are presented in Table 2. Due to the water loss, the cucumber surface luster diminished, and the lightness value decreased. The chlorophyll in cucumber skin was degraded with the extension of storage time (Li et al., 2021). Consequently, more yellow tones were observed in the peel of the control group. However, the increase in b* value of the coated group was lower than that of the control group, which indicated that the coating blocked the permeation of oxygen and thus slowed down the degradation of chlorophyll (Istúriz‐Zapata et al., 2020). Furthermore, the total color difference (ΔE) was lower in the coated group than in the control group after 15 days. Thus, the K_6_C_4_ coating played an effective role in maintaining the freshness of cucumbers.

**TABLE 2:** Changes in surface color of cucumbers during cold storage.

Storage time (days)	Control					Coating				
	L*	a*	b*	ΔE	Appearance	L*	a*	b*	ΔE	Appearance
0	49.32 ± 0.79^Aa^	−4.94 ± 0.54^Aa^	15.55 ± 0.44^Ca^	0 ^Da^	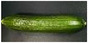	50.03 ± 1.50^Aa^	−5.25 ± 0.35^Aa^	14.99 ± 0.68^Ba^	0 ^Da^	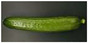
3	48.35 ± 1.19^Aa^	−4.79 ± 0.54^Aa^	16.21 ± 0.28^Ba^	1.23 ± 0.28^Ca^	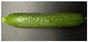	49.68 ± 1.87^Aa^	−4.98 ± 0.67^Aa^	15.07 ± 0.32^Ab^	0.91 ± 0.26^Ca^	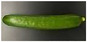
6	47.41 ± 1.19^Ba^	−4.51 ± 0.42^Aa^	16.63 ± 0.66^Ba^	2.29 ± 0.45^Ba^	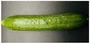	48.72 ± 2.14^Ba^	−4.96 ± 0.21^Aa^	15.06 ± 0.77^Ab^	1.40 ± 0.58^Ba^	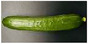
9	46.97 ± 0.33^Cb^	−4.53 ± 0.42^Aa^	16.83 ± 0.20^Aa^	2.87 ± 0.56^Ba^	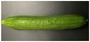	48.92 ± 0.70^Ba^	−4.72 ± 0.49^Aa^	15.81 ± 0.38^Ab^	1.74 ± 0.30^Ab^	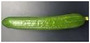
12	47.03 ± 0.94^Ba^	−4.17 ± 0.16^Ab^	17.16 ± 0.64^Aa^	3.11 ± 0.93^Aa^	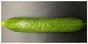	49.26 ± 1.78^Aa^	−4.70 ± 0.27^Aa^	15.54 ± 0.97^Ab^	1.34 ± 0.22^Bb^	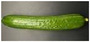
15	46.29 ± 0.75^Ca^	−3.99 ± 0.44^Ba^	17.75 ± 0.43^Aa^	3.93 ± 0.52^Aa^	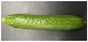	48.20 ± 0.89^Bb^	−4.57 ± 0.63^Ba^	16.08 ± 0.84^Ab^	2.51 ± 0.40^Ab^	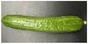

*Note*: Values are means ± standard deviation of three replicates. Different superscript uppercase letters (A–D) represent the significant differences within groups (*p* < 0.05), and different superscript lowercase letters (a–b) represent the significant differences between groups at the same time (*p* < 0.05).

#### 3.10.3 Total solid content and total acid

**FIGURE 6:**
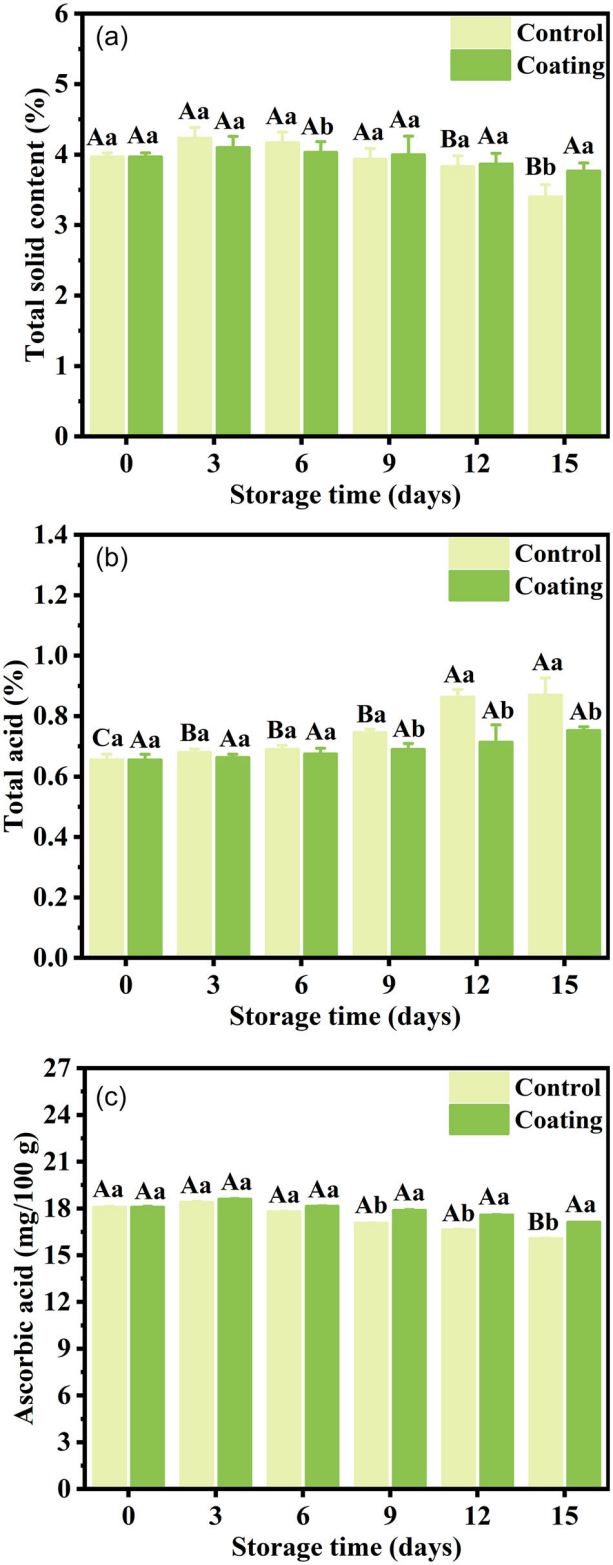
The total solid content (a), total acid (b), and ascorbic acid (c) of cucumbers during cold storage. Different uppercase letters (A–C) represent significant differences within groups (*p* < 0.05), and different lowercase letters (a–b) represent the significant differences between groups at the same time (*p* < 0.05).

Total solid content is an important parameter to evaluate the maturity and quality of fruits and vegetables. The aging of fruits and vegetables is often associated with a decline in total solid content (Chen et al., 2023c). As shown in Figure 6a, compared to the beginning, the total solids content of both the coated and control groups at the storage end showed a decreasing trend. This indicated that the cucumbers were aging. Total solids are the substances involved in carbohydrate metabolism in cells. At the late stage, the total solid content of the control group was 10.9% lower than the coating group. The results indicated that the K_6_C_4_ coating significantly delayed the decrease of total solid content at low temperature and inhibited respiration. When undergoing respiration, certain nutrients were consumed, leading to a decrease in total solid content (Riva et al., 2020).

Total acid content can reflect the ripeness of fruits and vegetables. The change in the total acid of cucumbers is presented in Figure 6b. The total acid of cucumbers showed a gradual increase during cold storage. In addition, the total acid of the control group was higher than that of the coated group. At the later stage of storage, the total acid content of the control group (0.87%) was significantly higher than that of the coated group (0.75%). The results indicated that organic acid anabolism had been carried out in cucumber pulp under the action of organic acid synthase, and coating treatment inhibited the organic acid synthesis but maintained the total acid level. The increase in total acid was mainly attributed to the conversion of sugars to organic acids (Anjum et al., 2020). The coating slowed down the loss of solids content and increase in total acid by inhibiting the respiration and other physiological metabolism of cucumbers.

#### 3.10.4 Ascorbic acid

In general, ascorbic acid content increases with fruit ripeness. After fruit ripening, ascorbic acid tends to decrease. It can be seen in Figure 6c that during the storage, the ascorbic acid content showed an increasing and then decreasing trend. It can be explained that the cucumbers still performed a series of physiological metabolisms after harvesting (Nunes, 2015). With the storage time increasing, the decomposition rate of ascorbic acid was accelerated. However, the ascorbic acid content of the coated group was 6.2% higher than that of the control group. Moreover, the rate of decline was faster in the control group compared to the coating group. These results suggested that the K_6_C_4_ coating significantly delayed the decrease of ascorbic acid content at low temperature and maintained it at a high level. This was mainly due to the barrier properties of the K_6_C_4_ coating and the inhibition against respiration. Similarly, the study reported that ascorbic acid content was well retained and decreased more slowly in cucumbers treated with chitosan coatings (Adeboyejo & Oyesanya, 2023). The barrier effect of the coating on gases (water vapor, oxygen, carbon dioxide, etc.) reduced the metabolic activity of the cucumbers and the oxidation of ascorbic acid, thereby contributing to prolonging the shelf life (Ghafoor et al., 2022).

#### 3.10.5 LF‐NMR relaxation and MRI of cucumbers

Table 3 shows the transverse relaxation time (*T*_2_) of cucumbers during cold storage. *T*_21_, *T*_22_, and *T*_23_ represented bound water, loosely bound water, and free water, respectively. Furthermore, *T*_21_, *T*_22_, and *T*_23_ also indicated the water in the cell wall, cytoplasm, and vacuole and extracellular, respectively (Donglu et al., 2016). In the control group and the coated group, the proportions of the three types of water showed a similar trend. In the beginning, free water was the dominant form in cucumbers that contained a small percentage of bound and loosely bound water. The free water increased and then decreased, with the opposite change of the bound and loosely bound water during the storage. This was mainly due to the water accumulation in respiration and the fact that the integrity of the cell membrane was damaged and the ability of fruit tissues to bind water was weakened, which led to the water migration from the bound to free. However, with the extension of storage time, the water was migrated back to the environment, resulting in a significant reduction in free water (Feng et al., 2018). Thus, the total water loss was mainly attributed to the loss of free water. In addition, the control samples lost more free water compared to the coated group, indicating that the K_6_C_4_ coating provided an effective water barrier for the preservation of cucumbers. This was consistent with the findings of Zhu et al. (Zhu et al., 2018). It was interesting to find that the bound and loosely bound water exhibited a significant increase, which might result from the conversion of partial free water or the respiratory metabolism.

**TABLE 3:** The water proportions (*A*_21_, *A*_22_, and *A*_23_) of cucumbers during cold storage.

	Control			Coating		
Storage time (days)	*A*_21_ (%)	*A*_22_ (%)	*A*_23_ (%)	*A*_21_ (%)	*A*_22_ (%)	*A*_23_ (%)
0	0.95±0.38Ca	6.39±0.71Ba	92.66±1.49Ba	0.95±0.38Ba	6.39±0.71Ba	92.66±1.49Ca
5	0.73±0.46Ca	1.99±0.86Ca	97.28±0.47Aa	0.86±0.22Ba	1.70±0.33 Da	97.44±0.51Aa
10	1.34±0.74Ba	6.45±0.66Ba	92.21±0.28Bb	0.84±0.27Bb	3.70±0.43Cb	95.46±0.63Ba
15	2.40±0.26Aa	23.37±0.48Aa	74.23±0.51Cb	2.44±0.28Aa	15.32±0.49Ab	82.24±0.35 Da

*Note*: Values are means ± standard deviation of three replicates. Different superscript uppercase letters (A–D) represent the significant differences within groups (*p* < 0.05), and different superscript lowercase letters (a–b) represent the significant differences between groups at the same time (*p* < 0.05).

**FIGURE 7:**
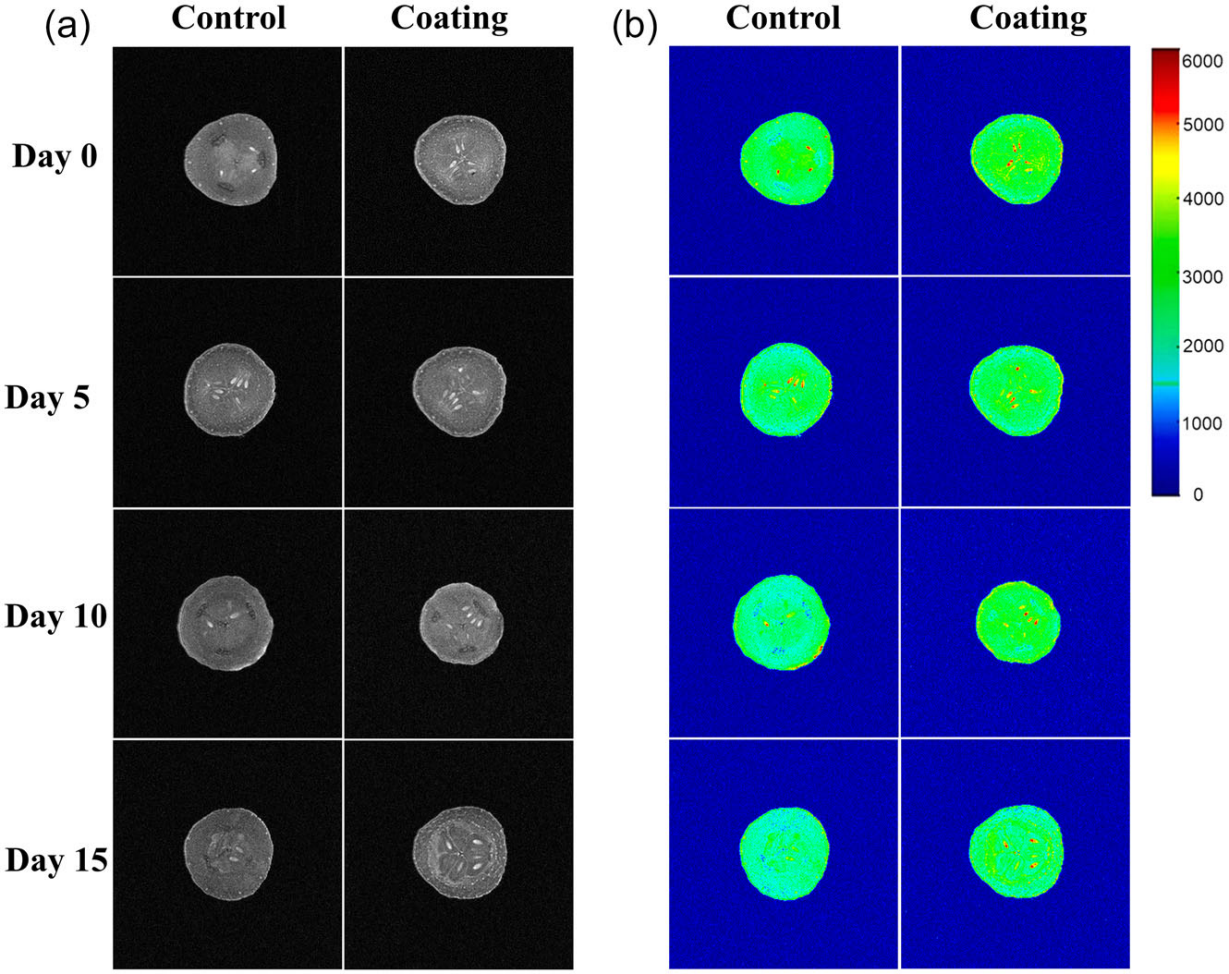
The magnetic resonance imaging (MRI) gray‐scale image (a) and pseudo‐color image (b) of cucumbers during cold storage.

Magnetic resonance imaging is a method of detecting the distribution of hydrogen‐containing substances in a sample by the density of hydrogen protons (Lagnika et al., 2013). The red color presents a high hydrogen proton density of water, and blue presents a low hydrogen proton density (Figure 7). Hydrogen protons in fruits and vegetables mainly originate from water molecules and sugar substances, which change during storage due to respiration and other physiological metabolism (Zhu et al., 2018). Figure 7 shows the MRI photographs of the coated cucumbers and the control during cold storage. The cross‐section of the cucumbers indicated that water was evenly distributed in the cucumber tissues, but it was noted that the protons in the seeds were high in signal intensity and the placenta was low in signal intensity at the storage beginning. It was observed that the area of water loss gradually increased, extending from the edge to the center. In the control group, the high signal intensity of the seeds gradually disappeared and moved to the edge (from day 5 to day 10), where there was no longer a high distribution of water at the end of storage. These results indicated that water was transferred from cucumber tissue to air continuously, and water in the center moved to the edge gradually. However, the seeds of the cucumbers with K_6_C_4_ coating maintained a high signal intensity throughout the cold storage, and the area showing low signal intensity was significantly smaller than that of the control group, and there was high signal intensity in the cucumber skin on day 10. These revealed that the 10th day might be the peak of water loss in cucumbers, and the K_6_C_4_ coating provided an excellent barrier, effectively reduced the transfer of water, and maintained the quality of cucumbers. Similarly, Fan et al. (2021) reported that the carbon‐dot/chitosan coatings could form a good barrier to reduce water loss, which exhibited a higher hydrogen proton density.

### 3.11 Inference of water transfer

**FIGURE 8:**
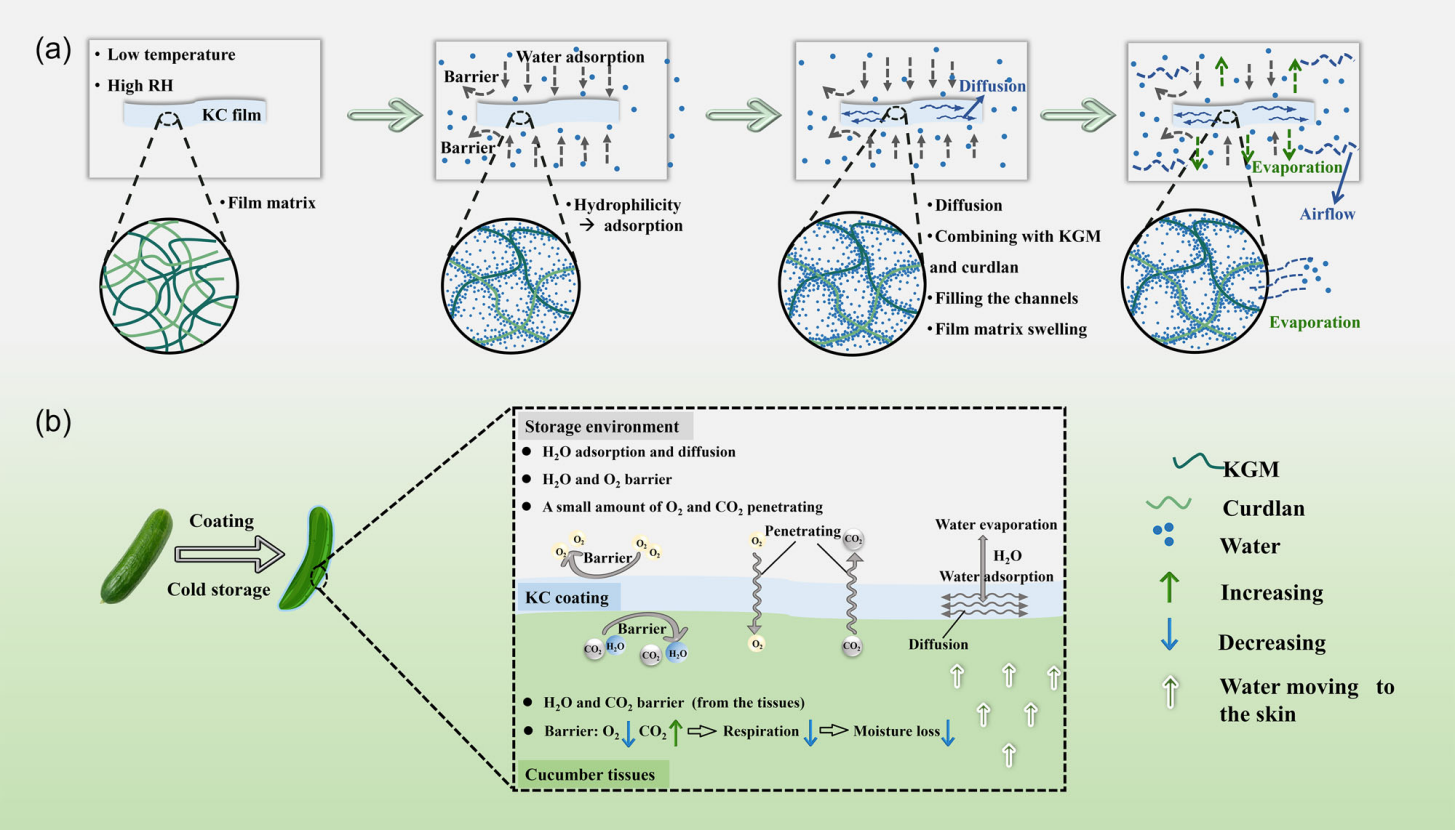
Inference of water transfer in konjac glucomannan (KGM)/curdlan (CUD) (KC) films (a) and KC‐coated cucumbers (b) at low temperature.

Moisture loss is a common and serious phenomenon in cold chain storage of fruits and vegetables, and water transfer is the mode of moisture loss. According to the results of KC films during cold storage, we can make an inference about the water transfer of the films. When KC films were exposed to low temperature and high RH, the films mainly showed an increase in weight, thickness, and moisture content increasing, and a decrease in gas barrier properties. The water in the surrounding air was adsorbed on the surface of the KC films, gradually diffused into the film matrix (Figure 8a), combined with KGM and curdlan molecules, and filled the intermolecular channel, making the film matrix stretch and the intermolecular channel smoother (Kuang et al., 2023). In addition, due to the existence of a certain air flow rate in the refrigerated environment, part of the water in the KC films will evaporate, and the water adsorption and evaporation occur cyclically, but the adsorption is greater than the evaporation, which depends on the hydrophilicity of the film skeleton molecules (Yan et al., 2023). As a carrier of low moisture content, the KC films mainly contained bound water, which did not change significantly during cold storage, mainly manifested as an increase of free water that came from the adsorption of water.

Once KC solution is used as a coating for cucumber preservation, the cold storage environment/KC coating/cucumbers (ECC) can be considered as a system. At low temperature, cucumbers maintained a low respiration rate and produced water (Ho et al., 2018). The free water in cucumbers gradually transferred from the center to the epidermis (Khan et al., 2018), and most of the water was blocked in cucumber tissues by KC coating (Figure 8b). In addition, the escape of carbon dioxide produced by cucumber and the entry of external oxygen was blocked by KC coating, so the respiration and moisture loss of cucumbers were inhibited (Ho et al., 2018). According to the results of the changes in film properties, it can be inferred that a small amount of water from cucumbers is absorbed and diffused by KC coating, and some of the water is transferred to the environment through KC coating. The water transfer between the cold storage environment and KC coating should be similar to the water transfer of KC films under cold storage.

## 4 CONCLUSION

After cold storage treatment, the KC films showed an increase in weight and thickness, and LF‐NMR revealed that the free water content of K_6_C_4_ film increased and the bound water content decreased, which was attributed to the water adsorption and diffusion of the KC films. The adsorbed water molecules played a plasticizing role, which increased the free volume among molecular chains, resulting in higher EAB value, lower temperature of endothermic peak, and larger ΔH of the endothermic peak for the film. K_6_C_4_ film appeared to be homogeneous and dense and exhibited the lowest soluble solid loss ratio and higher WCA. The addition of curdlan improved the barrier against H_2_O, O_2_, and CO_2_ and mechanical properties, and K_6_C_4_ retained gas barrier stability. The most stable matrix was formed in K_6_C_4_, showing excellent structural and properties stability. K_6_C_4_ coating significantly maintained the quality of cucumbers. In the ECC system, K_6_C_4_ coating inhibited the transfer of water from the center to the epidermis of cucumbers by blocking the water produced by respiration and the free water in its tissues. The storage stability of the films and water transfer analysis in this study will benefit the understanding of the mechanism of coating inhibiting fruit and vegetable moisture loss. In the future, there should be more consideration of developing multifunctional coating with the temperature and humidity of storage environment, characteristics, and respiratory metabolites of fruits and vegetables as a response indicator to more accurately and efficiently maintain the quality of fruits and vegetables.

## AUTHOR CONTRIBUTIONS

**Runmiao Tian**: Writing—original draft; formal analysis; methodology. **Jun Jiang**: Writing—review and editing; investigation. **Kao Wu**: Writing—review and editing. **Ying Kuang**: Writing—review and editing. **Bo Peng**: Writing—review and editing. **Kai Chen**: Methodology; writing—review and editing; resources. **Fatang Jiang**: Funding acquisition; writing—review and editing; resources; supervision.

## CONFLICT OF INTEREST STATEMENT

The authors declare no conflicts of interests.
